# Natural variation of the *GmDt1* gene affects the 100-seed weight of soybean

**DOI:** 10.3389/fpls.2026.1801920

**Published:** 2026-04-02

**Authors:** Guoji Wang, Duo Zhao, Xiaoyu Hu, Haowei Zheng, Wei Wang, Pengyu Bai, Long Miao, Huihui Gao, Longlong Wang, Hongye Sun, Jiajia Li

**Affiliations:** School of Agronomy, Anhui Agricultural University, Hefei, China

**Keywords:** 100-seed weight, BSA-seq, haplotype, soybean, variation

## Abstract

100-seed weight (100-SW) is a critical determinant of soybean yield. The identification and functional characterization of its underlying genes are therefore essential for the genetic improvement of seed and yield-related traits. A residual heterozygous line (RHL) segregating for 100-SW was derived from a recombinant inbred line (RIL) population generated by crossing small-seed (19.75 ± 1.93 g) and large-seed (26.20 ± 0.82 g) soybean parents. Phenotypic segregation of 100-SW was analyzed, and Chi-square test was used to verify the segregation ratio. Bulked segregant analysis combined with whole-genome sequencing (BSA-seq) was performed using both Euclidean distance and index algorithms to map the target gene. Functional annotation, molecular marker validation, and germplasm resequencing were conducted to identify the key candidate gene and its haplotypes. Phenotypic analysis showed significant segregation and normal distribution of 100-SW in the RHL, with a Chi-square-verified 1:2:1 segregation ratio, indicating control by a single nuclear gene. BSA-seq mapped the gene to a 5.46 Mb region on chromosome 19, where 74 non-synonymous SNPs in coding sequences were identified (including one causing initiation codon loss), distributed across 36 genes. *GmDt1* (*Glycine max Determinant stem 1*) was confirmed as the key candidate gene, with a G-to-T non-synonymous mutation in its first exon as the functional locus (validated in the original RIL population). Resequencing of diverse germplasm classified *GmDt1* into five haplotypes; the large-seed haplotype GmDt1-H2 was absent in wild soybeans, present in 9.07% of landraces, and 15.83% of cultivated soybeans. The gradual increase in the frequency of *GmDt1-H2* from wild to cultivated soybeans suggests that this haplotype has been positively selected during soybean breeding. Identification of *GmDt1* and its functional mutation provides a valuable molecular target for the genetic improvement of soybean seed traits and yield.

## 1 Introduction

As one of China’s major grain, oil, and cash crops, soybean provides a substantial source of plant protein and edible oil for human consumption, and also serves as a primary protein source for animal feed (Li et al., 2014; Miao et al., 2020). According to data from the National Bureau of Statistics of China, the average soybean yield in 2025 was 2039 kg per hectare, which is not only lower than the global average (approximately 2930 kg per hectare) but also far below that of major soybean-producing countries such as the United States and Brazil (approximately 3750 kg per hectare). Moreover, China’s large population exacerbates the imbalance between domestic supply and demand, resulting in heavy reliance on soybean imports. Consequently, improving soybean yield in China has thus become a pressing issue in Chinese agriculture. Soybean yield per unit area is determined by both planting density and individual plant productivity, with 100-seed weight (100-SW) being a key factor influencing the latter (Paran and Zamir, 2003). Therefore, research on soybean 100-SW holds important theoretical and practical significance.

During the domestication of wild soybeans into cultivated varieties, seed size has been progressively selected, with humans preferentially favoring seeds with higher 100-seed weight (Lee et al., 2011; Han et al., 2016). Seed size is a complex trait influenced by multiple genetic and environmental factors, with genetic factors playing a predominant regulatory role. Studies have reported that the broad-sense heritability of seed size can reach as high as 0.97 (Zhang et al., 2016; Yan et al., 2017). In soybean, several genes and loci have been identified as key regulators of seed size. For instance, the *POWR1* gene undergoes truncation in its CCT domain due to transposon insertion, resulting in increased 100-seed weight (Goettel et al., 2022). *GmSWEET10a/b* modulates seed size by transporting carbohydrates from the seed coat to the inner seed, and GmSWEET10a has been shown to interact with GmDt1 to coordinately regulate soybean seed size (Wang et al., 2020; Li X. et al., 2024). *GmSSS1* encoding a SPINDLY homolog that affects cell proliferation and division, thereby influencing 100-SW (Zhu et al., 2022). A T/C polymorphism at the *SW9–1* locus on chromosome 9 accounts for 10.05%-10.93% of the phenotypic variation in seed size (Li J. et al., 2019). *GmSMS6* interacts both physically and genetically with the transcription factor GmbZIP151 and the RING-type E3 ligase GmUBQ1; as a regulatory hub, it coordinates the transcriptional activation by GmbZIP151 and the GmUBQ1-mediated protein stability, and primarily repressing cell expansion in soybean seeds (Li et al., 2025). Natural variation in the promoter region of *GmST05* affects its transcriptional level, thereby regulating seed size (Duan et al., 2022). Several transcription factors including *GmMYB73*, *GmMYB181*, *GmBS1*, *GmBS2*, *GmWRKY15a*, and *GmSW14*, have been implicated in seed size regulation (Liu et al., 2014; Ge et al., 2016; Gu et al., 2017; Yang et al., 2018; Zhang et al., 2025) while substrate enzymes such as *GmFAD3*, *GmCYP78A10*, *GmCYP78A72*, *GmCYP78A5*, *GA20OX*, *GmCIF1*, *GmUBC1*, and *GmSW17* also contribute to the regulation of seed size (Singh et al., 2011; Wang J. et al., 2015; Lu et al., 2016; Zhao et al., 2016; Du et al., 2017; Tang et al., 2017; Mao et al., 2020; Liang et al., 2024).

Bulked segregant analysis (BSA) is a target gene mapping approach that involves pooling individuals with extreme phenotypes from segregating progeny within a population, followed by comparison of polymorphisms between the contrasting bulks and their associated phenotypes (Michelmore et al., 1991; Abe et al., 2012). Compared with traditional QTL mapping methods, BSA analysis of only a small subset of extreme individuals, thereby simplifying the experimental process and reducing costs (Yang et al., 2013). BSA-seq can be applied to various population types, including segregating genetic populations, natural populations, and multi-family populations. In agricultural research, segregating populations are most commonly used and include F_1_, F_2_ or F_2:3_-derived populations, backcross populations, RIL populations, and doubled haploid (DH) populations. Secondary segregating populations, such as chromosome segment substitution lines (CSSLs) and residual heterozygous lines (RHLs), are also suitable for BSA-based bulk analysis (Wang et al., 2023).

In this study, two major cultivated soybean cultivars ZP661 and ZH13 exhibiting significant differences in 100-SW were used to construct a mapping population. The candidate gene *GmDt1* was identified through BSA-based bulked pool sequencing, and further analysis revealed that a non-synonymous mutation in the first exon of *GmDt1* serves as the functional locus regulating 100-SW. This study thus provides a valuable molecular target for the improvement of soybean yield.

## 2 Materials and methods

### 2.1 Experimental materials

Two soybean cultivars ZP661 (100-SW: 19.75 g) and ZH13 (100-SW: 26.20 g) were used in this study. These parental lines were crossed in 2019 to produce F_1_ progeny, which were subsequently self-pollinated to generate F_2_ individuals in 2020. A RIL population was ultimately established after ten successive generations of selfing. From this population, a RHL exhibiting significant phenotypic segregation for 100-SW was identified (**Supplementary Figure 1**). To minimize environmental variation, the experimental materials were planted at two locations, Suzhou and Mingguang, in Anhui Province, China. All materials were arranged in a randomized complete block design (RCBD) and managed according to standard field practices. The 100-SW of each line was measured at soybean maturity.

### 2.2 Phenotypic and genetic analysis

Phenotypic evaluation of 100-SW in RHLs and RILs was conducted as follows: 100 seeds were randomly selected from each line, and their total weight was measured using an electronic balance.

Individual plants in the segregating RIL population were tagged and sampled at the R6 growth stage. Following harvest, the 100-SW data of mature plants were used to perform a chi-square test to determine the genetic inheritance of this trait. The analysis was carried out using the following formula:


$$\chi^{2}=\sum_{i=1}^{k}\frac{{\left(O_{i}-E_{i}\right)}^{2}}{E_{i}}$$

*O_i_* represents the observed number of plants; *E_i_* represents the expected number of plants, *k* represents the total number of categories.

### 2.3 BSA-Seq based candidate interval mapping

Based on the phenotypic traits of the two parental lines and the RHLs, four DNA pools were constructed: two parental pools, a small-seed bulk (Bulk A), and a large-seed bulk (Bulk B). Each parental pool consisted of 10 individual plants from ZP661 or ZH13, respectively. For the progeny bulks, 30 ZP661-like plants and 30 ZH13-like plants were selected from the segregating RHL population, and leaf samples from these plants were pooled to construct the two progeny bulks. Sequencing was performed at a depth of 30× for the progeny bulks and 10× for the parental pools. The experimental procedure was as follows: young top leaves were collected from each selected plant, and genomic DNA was extracted from individual leaf samples using the CTAB method. After assessing DNA quality and quantifying DNA concentration, equal amounts of DNA from each plant within a pool were combined to generate the four bulks. Subsequent sequencing and bioinformatic analyses were performed by Biomarker Technologies Corporation (Beijing, China).

To ensure the quality of subsequent analyses, raw sequencing reads generated by the Illumina platform were stringently filtered to obtain high-quality clean reads. All data processing followed a standardized workflow, with the software and specific steps described below. The processed data were subsequently used for alignment variant detection, and other downstream analyses.

FastQC (v0.11.9) was used for preliminary quality assessment of the raw sequencing data. This included evaluating sequence quality distribution, base composition, adapter contamination, and other metrics. Trimmomatic (v0.39) was employed to remove low-quality reads, adapter sequences, and abnormal sequences; Samtools (v1.9) was used for format conversion, sorting of alignment results, and related statistical analyses. MultiQC (v1.14) was applied to visualize FastQC results and integrate data quality control reports.

Raw reads were filtered using Trimmomatic software with the following core parameter settings: LEADING: 3, TRAILING: 3, SLIDINGWINDOW: 4: 15, MINLEN: 100. The specific filtering criteria are described below, fully consistent with the subsequent results section. Reads containing adapter sequences were removed using the built-in Illumina adapter library. After adapter removal, paired-end reads shorter than 100 bp were filtered out. Paired-end reads longer than 100 bp were retained for subsequent analysis.

Filtering of reads with high N content: The proportion of unrecognized bases (N) in each read was calculated. Reads with an N content exceeding 10%, along with their paired-end sequences were filtered out. Removal of low-quality reads: A base quality threshold of Q10 corresponding to a sequencing error rate of 10%, was applied. If more than 50% of the bases in a read had a quality value below 10, the read and its paired-end sequence were discarded.

Euclidean Distance (ED) algorithm for association analysis: This method uses sequencing data to identify markers showing significant differences between bulks, thereby pinpointing genomic regions associated with a trait. In theory, loci not associated with the target trait should show little to no difference between the two bulks, while loci linked to the target trait will display differences. Consequently, ED values for non-target loci are expected to approach 0. Larger ED values indicate greater differences in marker allele frequencies between the two bulks. The ED is calculated using the following formula, where “mut” denotes the allele frequency in the mutant bulk, and “Wt” denotes the allele frequency in the wild-type bulk.


$$ED=\sqrt{{\left(\text{Amut}-\text{AWt}\right)}^{2}+{\left(\text{Cmut}-\text{CWt}\right)}^{2}+{\left(\text{Gmut}-\text{GWt}\right)}^{2}+{\left(\text{Tmut}-\text{TWt}\right)}^{2}}$$

Index association analysis is a marker-trait association method based on differences in genotype frequencies between bulks. It identifies significant variations in genotype frequencies and quantifies these differences using the Δindex metric. The calculation formula is as follows, where “aa” represents the wild-type progeny bulk, “ab” represents the mutant progeny bulk, and “M” and “P” denote the read counts of alleles derived from the wild-type parent and the mutant parent in their respective bulks. The Δindex allows the assessment of differences at each locus between the mutant and wild-type bulks. Specifically, the stronger the association between a marker and the target trait, the closer the Δindex value is to 1.


Index (aa)=Maa/(Maa+Paa)

Index (ab)=Mab/(Mab+Pab)

Δindex=Index (aa)-Index (ab)

### 2.4 Alignment of sequencing reads, annotation of variants and haplotype analysis

Sequencing reads were aligned, and detected variants (e.g., the variant located 183 bp downstream of the ATG initiation codon of *GmDt1*) were annotated using the soybean reference genome (*Glycine max* Wm82.a4.v1) and the corresponding gene annotation files. These resources were downloaded from the Phytozome database (https://phytozome-next.jgi.doe.gov/), a comprehensive plant genome repository hosted by the US Department of Energy Joint Genome Institute (JGI). Haplotype analysis of variant sites was conducted using the Soybean Genomic Variation Database (SoyGVD, https://yanglab.hzau.edu.cn/SoyGVD/#/variation).

### 2.5 RNA extraction and reverse transcription from plant tissues

Total RNA was extracted from soybean tissues using the TaKaRa MiniBEST Plant RNA Extraction Kit (TaKaRa, Dalian, China). RNA integrity was verified by electrophoresis, and only samples showing clear 28S and 18S ribosomal RNA bands were used for subsequent experiments. RNA concentration was then measured using a micro-spectrophotometer, and the samples were stored at -80 °C until use. First-strand cDNA was synthesized using the PrimeScript™ RT. Reagent Kit with gDNA Eraser (Perfect Real Time) (TaKaRa, Dalian, China) following the manufacturer’s instructions.

### 2.6 Primer design

The soybean reference genome from the Phytozome database (https://phytozome-next.jgi.doe.gov/) was used to retrieve the target amplicon sequences. Specific primers were designed based on these sequences using Primer 5.0 software. The primers used in this study are listed in **Table 1**.

**Table 1:** Primers used in this study.

Primer names	Primer sequence 5’-3’
dCAPS-19g181900-F	ATGCTATCAGATGTAGGGATGAACCAGAGTTACTTCAAAATGTAAACC
dCAPS-19g181900-R	GAATCTTGGCTTCTTTTGTTGTGTTGAG
*Dt1*-frag1-F	GGGGCAAAACACACTCGAT
*Dt1*-frag1-R	AGTTCTGTAATGTTTGTTTGAGACT
*Dt1*-frag2-F	GTTTCTCTTAATAACTTAACCTCTT
*Dt1*-frag2-R	TACTACAGAACGTACACAACATCT
*qDt1-F*	TGTTCCTCCTCTTACAATGGCA
q*Dt1*-R	GCTTCTTGTTGTAACTCACAGTC

### 2.7 Development of dCAPS molecular markers

The dCAPS markers were developed using SnapGene software. PCR amplification was performed on the selected polymorphic loci using Taq-Master DNA polymerase. The specific PCR reaction system (15 μL total volume) and conditions are detailed as follows: PCR reaction system (15 μL): ddH_2_O 5.3 μL, Taq Master Mix 7.5 μL, forward/reverse primers (F/R) 0.6 μL each, and genomic DNA 1 μL. PCR reaction conditions: pre-denaturation at 95 °C for 5 min; followed by 36 cycles of denaturation at 98 °C for 30 s, annealing at 56 °C for 30 s, and extension at 72 °C for 45 s; followed by a final extension at 72 °C for 5 min. PCR products were analyzed by 1.5% agarose gel electrophoresis to verify the presence of a single target band. Qualified PCR products were then digested with the appropriate restriction endonuclease for 8 hours The fragment lengths of the digested products were determined by 2.5% agarose gel electrophoresis.

### 2.8 Expression analysis of *GmDt1*

*GmTubulin* was used as the reference gene for quantitative real-time PCR (qRT-PCR) normalization. The qRT-PCR assay was performed using AceQ Universal SYBR qPCR Master Mix. which contains the fluorescent SYBR Green I DNA-binding dye (Vazyme, Nanjing, China). The assay was conducted away from direct strong light to prevent dye degradation. The total reaction volume was 20 μL, consisting of 10 μL of 2× AceQ Universal SYBR qPCR Master Mix, 7.2 μL of ddH_2_O, 0.4 μL each of forward and reverse primers, and 2 μL of cDNA template. Each reaction was performed in three biological replicates to ensure reproducibility.

### 2.9 Statistical analysis

All experimental data were obtained from three or more independent replicate experiments and analyzed using Student’s t-test. Data collation and statistical analyses were performed using Microsoft Excel. Graphs were generated using GraphPad Prism 8 software. A statistically significant difference between two data groups was defined at the 95% confidence level.

## 3 Results

### 3.1 Phenotypic identification and genetic analysis of 100-SW in residual heterozygous lines

**Figure 1:**
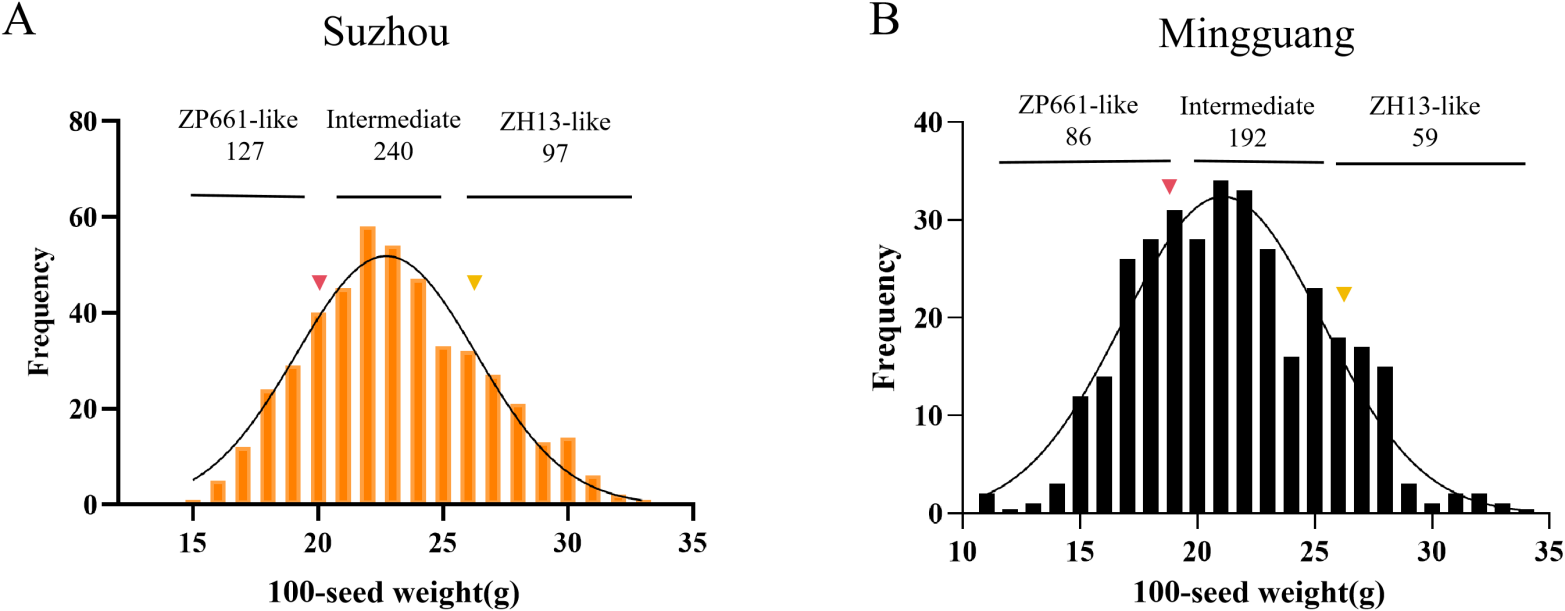
Characteristics of 100-SW distribution in residual heterozygous lines. **(A)** Distribution of 100-seed weight in recombinant heterozygous lines grown in Suzhou, Anhui Province, ranging from 15.21 to 32.72 g (n = 464). **(B)** Distribution of 100-seed weight in recombinant heterozygous lines grown in Mingguang City, Anhui Province, ranging from 11.56 to 33.08 g (n = 337). Red triangles indicate the 100-SW of ZP661, and yellow triangles indicate the 100-SW of ZH13. Each individual plant’s 100-seed weight was measured in three technical replicates.

In the previously constructed F_2_:_10_ RIL population derived from a cross between ZP661 (100-SW: 19.75 ± 1.93 g) and ZH13 (100-SW: 26.20 ± 0.82 g), a segregating RHL was identified. This RHL line was planted in Suzhou and Mingguang, Anhui Province, where significant segregation of the 100-SW trait was observed among the segregating individuals. The 100-SW values of these plants exhibited a normal distribution at both locations (**Figure 1**). The mean 100-SW was 23.12 ± 3.47 g with a coefficient of variation (CV) of 14.98% in Suzhou, whereas the mean 100-SW in Mingguang was 21.42 ± 3.94 g with a CV of 18.39%. Due to the larger sample size and more stable genetic variation at the Suzhou site, phenotypic characterization was performed on 464 individual plants of this RHL line grown in Suzhou for subsequent genetic studies. Chi-square test results revealed that the phenotypic segregation ratio fitted 1:2:1 (*P* > 0.05), indicating that 100-SW in this population is likely controlled by a single major gene (**Figure 1A**, **Table 2**).

**Table 2:** Genetic analysis of 100-SW in soybean.

Plant materials	Sum	Expected value	Observed value	Expected ratio	*χ^2^*	*P-*value
ZP661-like	464	116	127	1:2:1	4.43	0.11
Intermediate		232	240			
ZH13-like		116	97			

### 3.2 Candidate gene mapping based on BSA bulked sequencing

#### 3.2.1 Data quality assessment

**Figure 2:**
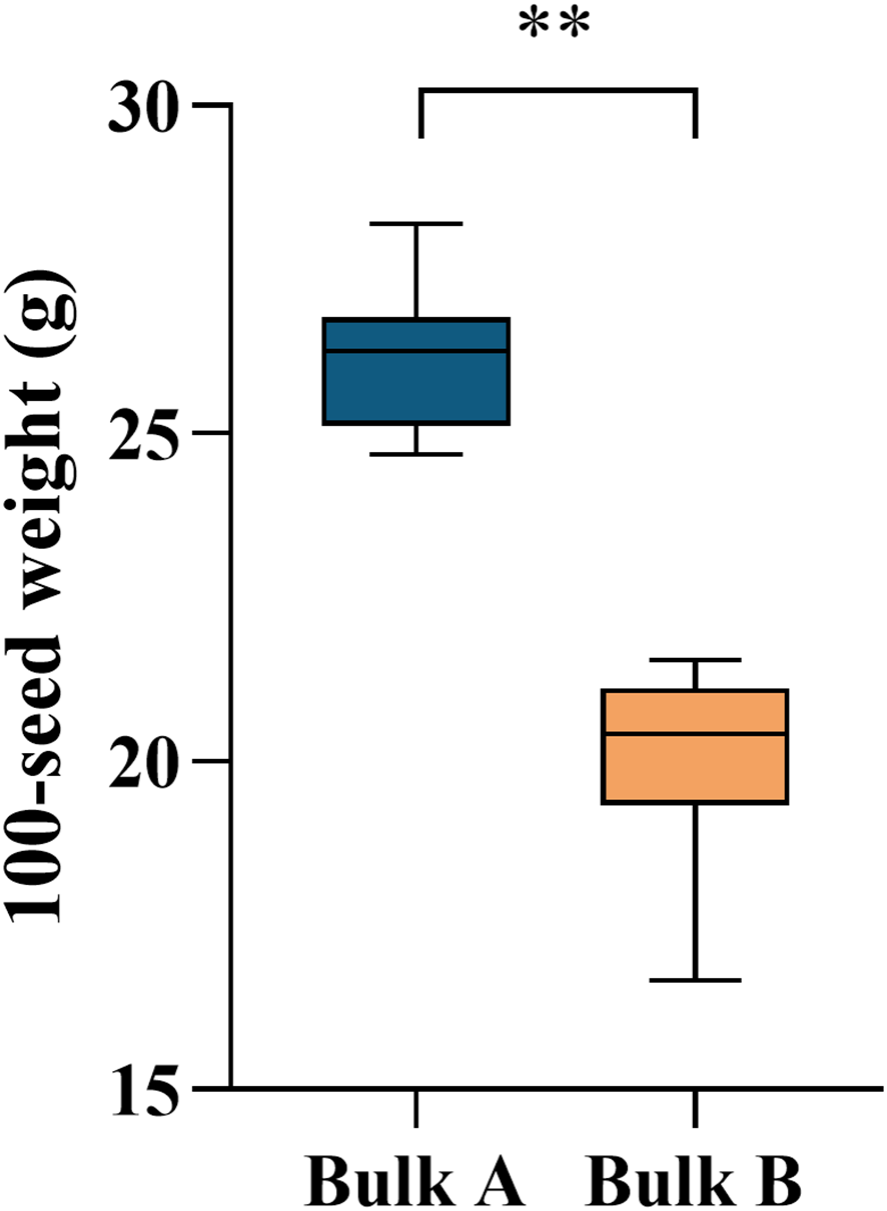
Average 100-SW of two progeny gene pools, Bulks A and B. Bulk A represents the large-seed weight pool, and Bulk B represents the small-seed weight pool. Data are shown as mean ± SEM (n = 30). Statistical significance was assessed using Student’s t-test (P< 0.01). ** indicates statistical significance at P < 0.01.

To further refine the mapping of candidate genes, two extreme bulks (Bulk A and Bulk B) were constructed. The average 100-SWs of Bulk A and Bulk B were 26.11 ± 1.12 g and 19.92 ± 1.38 g, respectively, showing an extremely significant difference (**Figure 2**). Candidate gene mapping was conducted using the BSA-based bulked sequencing approach. Whole-genome resequencing was performed on the four samples, followed by data filtering. Reads containing adapters were removed. Reads with an N content exceeding 10% were discarded. Reads in which more than 50% of bases had a quality score below 10 were also excluded. The final results indicated that the Q30 value exceeded 80%, demonstrating that the sequencing data were of sufficient quality for subsequent analyses (**Table 3**).

**Table 3:** The QC data of the whole genome sequencing.

Library ID	Clean_reads	Clean_base	Q20 (%)	Q30 (%)	GC (%)
Bulks A	201945448	30157281558	99.58	96.97	35.79
Bulks B	218916416	32690298970	99.56	96.76	36.01
ZP661	91995608	13733918996	99.57	96.92	35.39
ZH13	97118664	14499465106	99.59	97.02	35.56

#### 3.2.2 Association analysis

**Figure 3:**
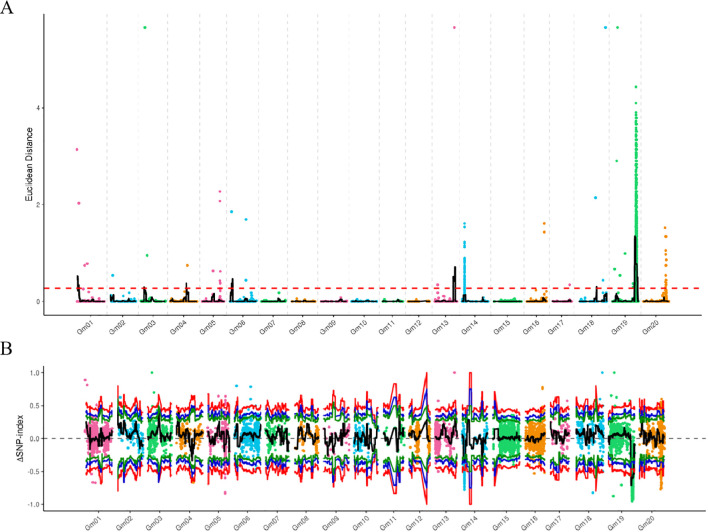
The ED and index correlation analysis of SNPs. **(A)** ED association analysis of SNPs. The x-axis represents chromosomal positions, and the y-axis denotes ED values. The black line shows the fitted ED association values, and the red dashed line indicates the significance threshold. **(B)** Index association analysis of SNPs. The x-axis represents chromosomal positions, and the y-axis denotes index values. The black line shows the fitted index association values, and the red line indicates the significance threshold.

Association analysis of these high-quality SNPs was conducted using two algorithms: the ED algorithm and the Index algorithm. Results showed that the ED algorithm-based SNP association analysis identified mapping intervals on chromosomes 1, 3, 4, 6, 13, and 19, with respective sizes of 3.66 Mb, 0.58 Mb, 0.18 Mb, 1.62 Mb, 3.98 Mb, and 6.06 Mb (**Figure 3A**, **Table 3**). In comparison, the Index algorithm-based SNP association analysis identified a single 5.46 Mb mapping interval on chromosome 19 (**Figure 3B**, **Table 4**). The intersection of results from the two association analysis methods defined a final 5.46 Mb candidate region on chromosome 19 (**Table 4**).

**Table 4:** Associated region on chromosome 19 based on SNP analysis.

Association analysis type	Chromosome ID	Start position	End position	Region size (Mb)
SNP correlates results (ED)	Gm01	0	2000000	2
	Gm01	2100000	3720000	1.62
	Gm01	3780000	3820000	0.04
	Gm03	6400000	6980000	0.58
	Gm04	30040000	30220000	0.18
	Gm06	4840000	6460,000	1.62
	Gm13	34900000	38880000	3.98
	Gm19	42660000	48720000	6.06
SNP correlates results (SNP-index)	Gm19	43240000	48700000	5.46
The intersection of association results	Gm19	43240000	48700000	5.46

#### 3.2.3 Mining of candidate genes

**Figure 4:**
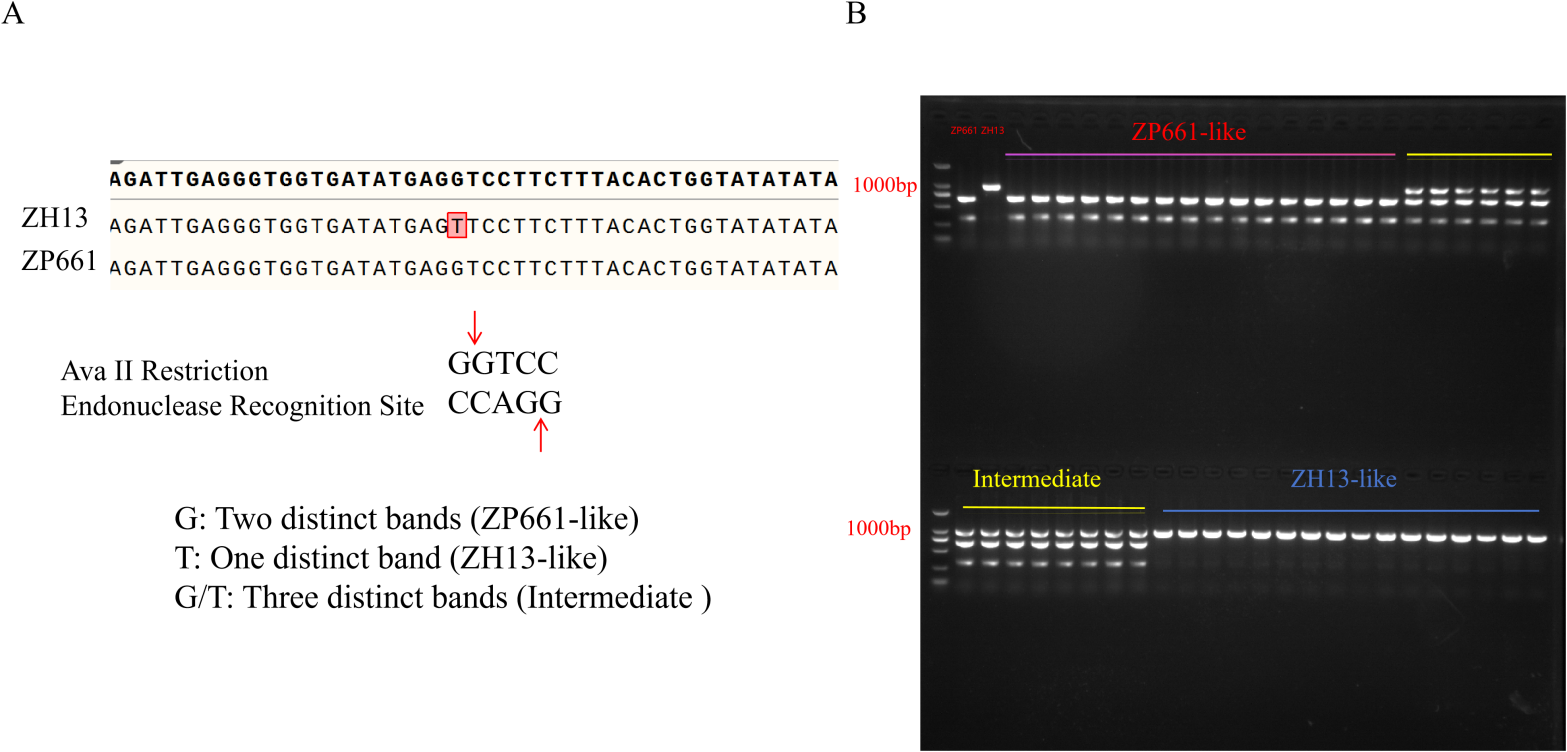
Molecular marker validation of the candidate gene *GmDt*1. **(A)** Schematic of the restriction endonuclease site identified by the CAPS marker. The red vertical line indicates the core restriction site. The ZP661 genotype can be digested by AvaII, whereas the mutated ZH13 genotype cannot. **(B)** CAPS marker electrophoresis profile of the segregating population. Two bands represent the ZP661 genotype, one band represents the ZH13 genotype, and three bands indicate the heterozygous genotype. The DNA marker is 2000 bp.

Analysis of variant sites within the 5.46 Mb candidate interval identified 74 single-nucleotide polymorphisms (SNPs) that induced non-synonymous mutations in genes and one SNP that resulted in the loss of the start codon. These functional variants were distributed across the coding regions of 36 genes (**Supplementary Table 1**). We analyzed the expression patterns and predicted biological functions of these 36 genes, and excluded those specifically expressed in tissues such as roots, flowers, and leaves. Finally, two auxin response factors, *Glyma.19G181900* and *Glyma.19G206100*, as well as one pod setting habit regulatory gene *Glyma.19G194300* (*GmDt1*, an ortholog of *Arabidopsis TFL1*), were identified as candidate genes underlying the target phenotype (**Table 5**). Further analysis of the BSA sequencing results for these three putative candidate genes showed that *Glyma.19G194300* and *Glyma.19G181900* exhibited relatively high ED and Δindex values. Therefore, these two genes were selected for subsequent investigation. First, validation analysis was conducted on *Glyma.19G181900*. A dCAPS molecular marker was developed based on the variant site to examine whether the phenotype of segregated progeny was tightly linked to this variation (**Supplementary Figure 2A**). The results showed that among 19 ZP661-like and intermediate individuals, one plant exhibited no linkage between the variation and phenotype. Among 15 ZH13-like individuals, three plants showed unlinked variation and phenotype (**Supplementary Figure 2B**). These results excluded *Glyma.19G181900* as a regulator of 100-SW. Subsequently, CAPS markers and sequencing were used to perform a correlation analysis between genetic variations in the *GmDt1* gene and phenotypic traits. The results revealed complete co-segregation between the variation and phenotype. Individuals with the T/T genotype had a 100-SW ranging from 24.68 to 28.20 g; those with the G/G genotype ranged from 16.66 to 21.54 g; and those with the G/T genotype ranged from 22.83 to 24.85 g (**Figure 4**; **Supplementary Table 2**). These results indicate that *GmDt1* is a candidate gene regulating 100-SW in soybean, with the functional variant located at position 183 downstream of the ATG initiation codon.

**Table 5:** Analysis of candidate gene variation loci.

Gene	Site	Variant base and amino acid	ED	Delta-index
*Glyma.19g194300* (*Gm*Dt1)	45625529 (1st exon)	AGG->AGT, R->S	1.018	0.720
*Glyma.19G181900*	44489593 (3rd exon)	AGG->AAG, R->K	0.997	0.705
*Glyma.19G206100*	46699857 (3rd exon)	ATG->TTG, M->L	0.893	0.631

#### 3.3 Analysis of relative expression level of *GmDt1* in parents

**Figure 5:**
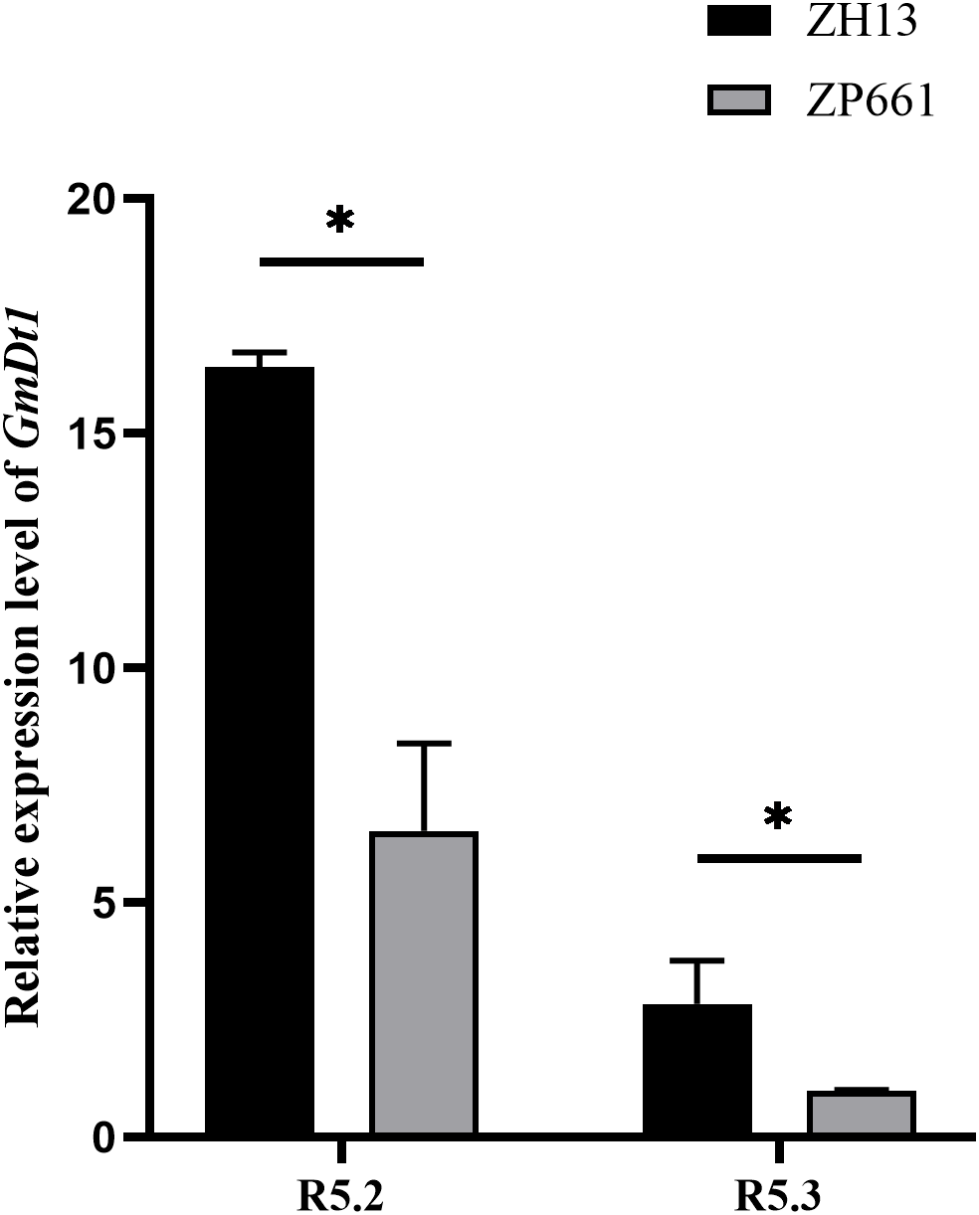
Relative expression analysis of *GmDt1* in parental lines. R5.2: seed length is approximately one-third of the pod cavity length. R5.3: seed length is approximately one-half of the pod cavity length. Data are shown as the mean ± SEM (n = 3). Statistical significance was determined using Student’s t-test (P< 0.05). * indicates statistical significance at P < 0.05.

To verify the differential expression of *GmDt1* in seeds of the parental lines, total RNA was extracted from seeds of ZH13 and ZP661 at the R5.2 and R5.3 stages, cDNA was then synthesized for quantitative real-time PCR analysis. The results showed that the expression level of *GmDt1* in ZH13 was significantly higher than that in ZP661 at both developmental stages (**Figure 5**).

#### 3.4 Analysis of 100-SW among different allelic types of *GmDt1*

**Figure 6:**
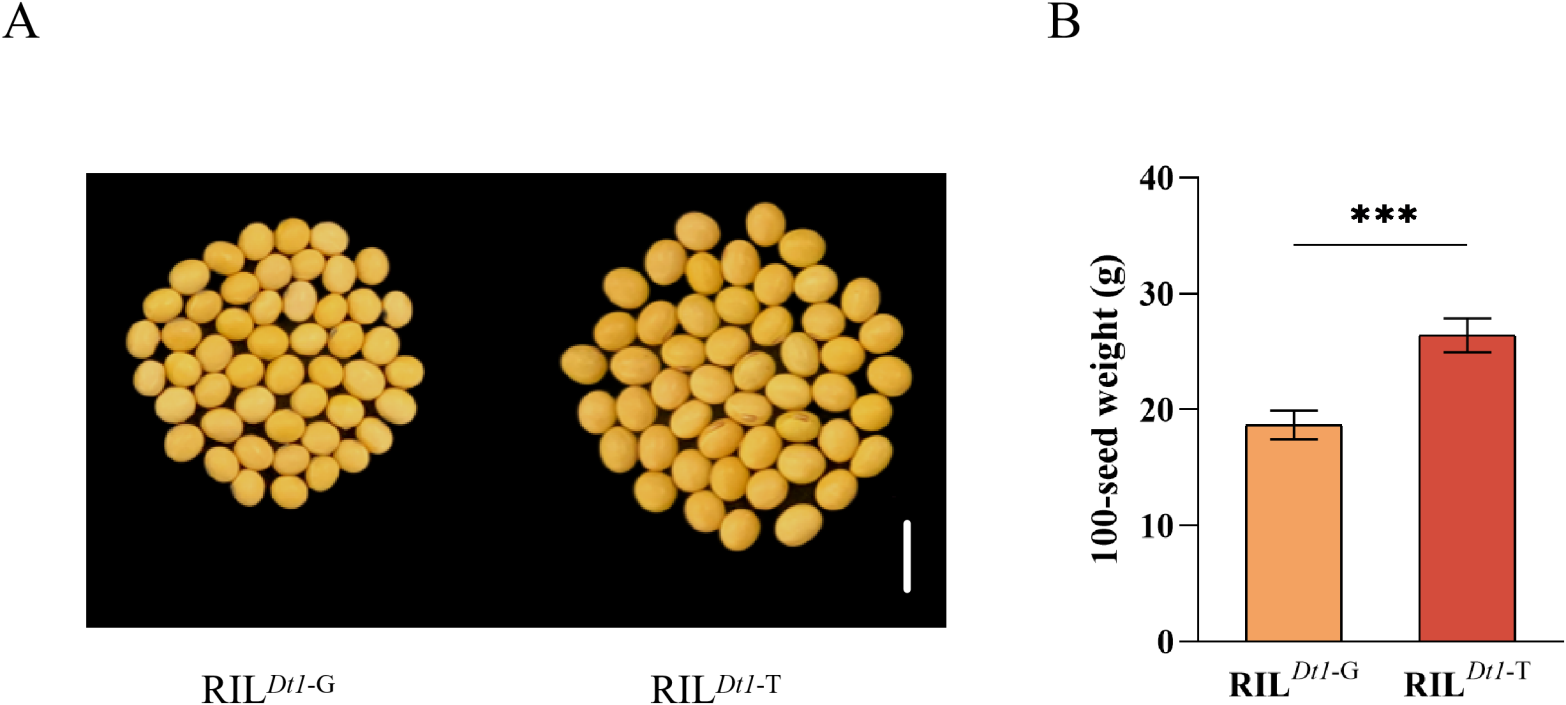
Comparison of differences in 100-SW between recombinant inbred lines RIL*^Dt1^*^-G^ and RIL*^Dt1^*^-T^. **(A)** Schematic diagram of grain size for RIL*^Dt1-G^* and RIL*^Dt1-T^* genotypes; the scale bar is 1 cm. **(B)** Grain weight of RIL*^Dt1-G^* and RIL*^Dt1-T^* genotypes in the RHL population; data are presented as the mean ± SEM (n = 40). Statistical significance was determined by Student’s t-test, with P< 0.001. *** indicates statistical significance at P < 0.001.

To further verify the effect of *GmDt1* alleles on 100-SW, sequencing was used to identify the *GmDt1-G* and *GmDt1-T* alleles in the RIL population derived from ZH13 and ZP661. Forty lines carrying each allele were randomly selected to compare their 100-SW values. The results showed that the average 100-SW of RIL*^Dt1-G^* was 18.68 ± 1.21 g, whereas that of RIL*^Dt1-T^* was 26.42 ± 1.46 g. This represents a significant increase of 41.45% in RILDt1-T compared with RILDt1-G (**Figure 6**).

#### 3.5 *GmDt1* haplotype analysis

**Figure 7:**
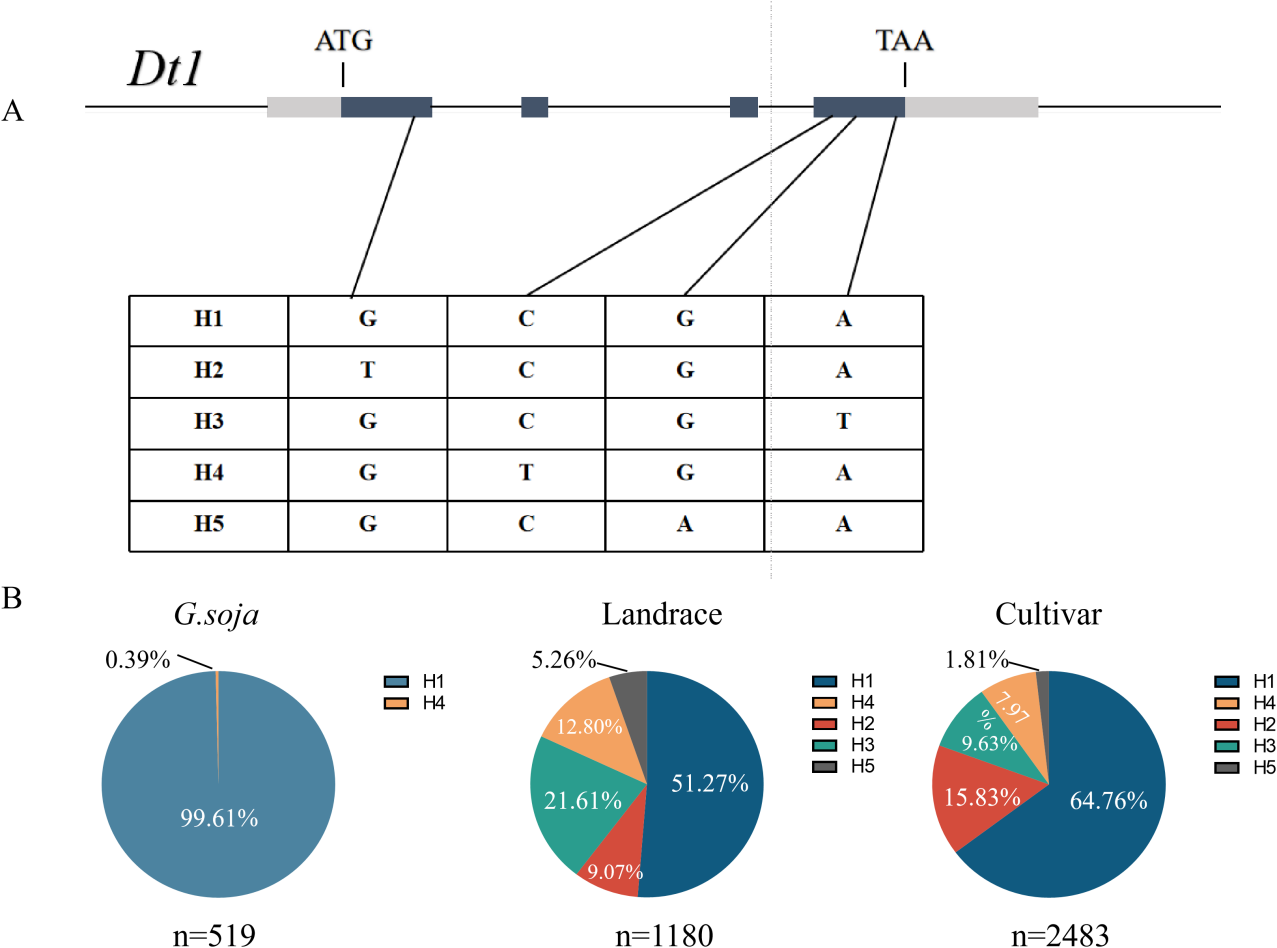
Haplotype and evolutionary analysis of *GmDt1*. **(A)** Haplotype structure of the *GmDt1* gene. Black boxes indicate exons, lines indicate introns, and gray boxes indicate UTRs. H1-H5 represent five distinct haplotypes. **(B)** Frequency distribution of the different *GmDt1* haplotypes in *G. soja*, landraces, and cultivars.

To investigate whether *GmDt1* alleles have been exploited during soybean domestication and improvement, genotyping of *GmDt1* was performed using resequencing data from public databases. A total of four non-synonymous mutations were identified in the coding sequence (CDS) region, which were classified into five haplotypes (**Figure 7A**). The parental lines ZP661 and ZH13 belonged to haplotypes H1 and H2, respectively. Analysis of the evolutionary characteristics of these five haplotypes revealed that wild soybean (*G. soja*) predominantly harbored the H1 haplotype (99.61%), with a small proportion of the H4 haplotype (0.39%). No other haplotypes were detected in wild soybeans. In contrast, three additional haplotypes (H2, H3, and H5) emerged in landraces and improved cultivars.

**Figure 8:**
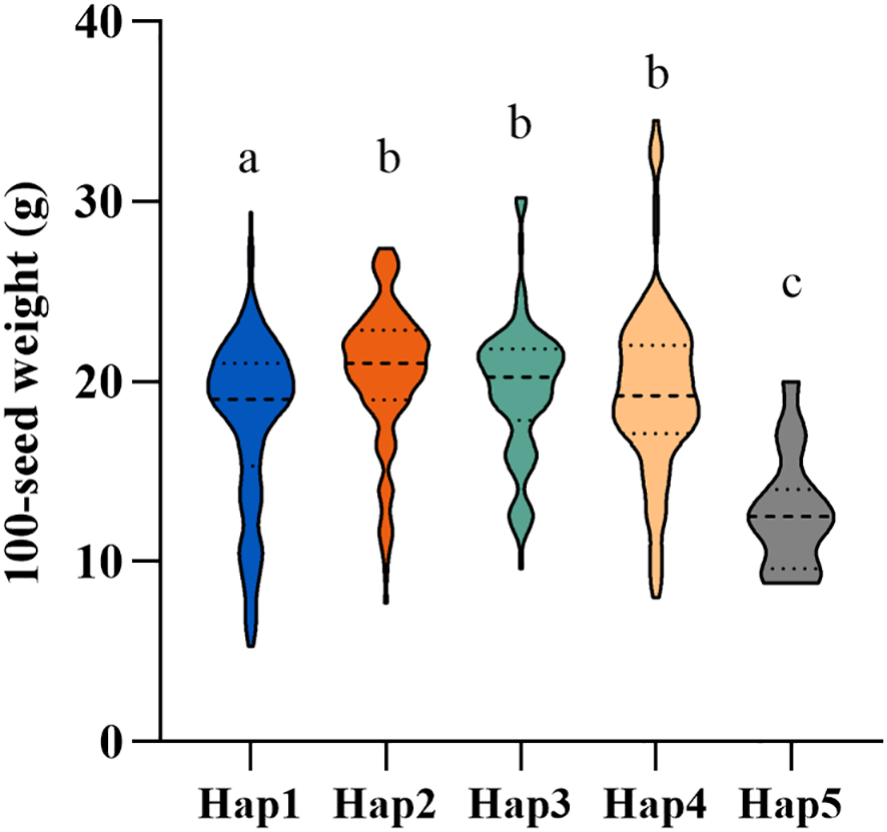
100-seed weight variation among five haplotypes (Hap1–Hap5) of *GmDt1*. The violin plot shows the distribution of 100-seed weight (g) for each haplotype, with sample sizes as follows: Hap1 (n = 150), Hap2 (n = 138), Hap3 (n = 99), Hap4 (n = 121), and Hap5 (n = 15). The dashed horizontal lines within each violin indicate the median. The upper and lower bounds of the central box represent the 25th and 75th percentiles, respectively. Different lowercase letters (a–c) above the violins indicate statistically significant differences between haplotypes (P< 0.05).

A total of 523 soybean accessions with clear provenance were randomly selected to analyze the differences in 100-seed weight among various haplotypes of *GmDt1*. The results showed that Hap2 exhibited the highest mean 100-SW (20.46 ± 3.85 g), which was significantly higher than that of Hap1 (17.83 ± 4.72 g) (**Figure 8**). Moreover, the proportion of H2, the haplotype associated with high 100-SW identified in this study, continuously increased from landraces to improved cultivars, rising from 9.07% to 15.83% (**Figure 7B**). These results indicate that the *GmDt1-H2* haplotype related to high 100-SW may have been gradually selected by breeders during soybean improvement.

To further investigate whether the gradual selection of the *GmDt1-H2* haplotype was driven by 100-SW rather than stem growth habit, we selected 165 released soybean cultivars carrying this haplotype. These accessions included lines from the Northern ecological zone, Huang-Huai-Hai ecological zone, and Southern ecological zone in China. Their stem growth habits were examined. The results showed that 130 accessions were determinate, 31 were semi-determinate, and 4 were indeterminate (**Supplementary Table 3**). These findings further confirm that the *GmDT1-H2* haplotype carrying *GmDt1-T* was selected during breeding primarily for high 100-SW, rather than for the determinate stem growth habit.

## 4 Discussion

### 4.1 Phenotypic variation in 100-SW of soybean

As a major oilseed and cash crop, soybean provides humans and livestock with abundant nutrients, including proteins and fats. In recent years, although global soybean yield has continued to increase, projections indicate that soybean production will need to double by 2050 to meet the growing demand (Jeong et al., 2012; Bodirsky et al., 2015). 100-SW is a key determinant of soybean yield. Developing soybean cultivars with an optimal 100-SW has thus become one of the primary objectives of plant breeders (Chen et al., 2023; Kumar et al., 2023; Xu et al., 2023). Notably, 100-SW in soybean is a quantitative trait controlled by multiple genes (Yan et al., 2017; Li X. et al., 2019). Therefore, identifying key genes regulating 100-SW will not only facilitate soybean yield improvement but also provide molecular targets for high-yield breeding programs.

In this study, RHLs derived from the cross between the small-seeded cultivar ZP661 and the large-seeded cultivar ZH13 were used to examine phenotypic variation and perform genetic analysis of soybean 100-SW. The 100-SW of these materials varied widely, ranging from approximately 15 g to 33 g, and followed a normal distribution. Previous studies have shown that the 100-SW of cultivated soybean varieties is roughly six times that of wild soybeans, reflecting substantial genetic variation (Zhang et al., 2016; Yu et al., 2017; Zhao et al., 2019). Consistent with these reports, the RHL population in this study also exhibited significant genetic variation, making it suitable for further genetic analysis.

Genetic analysis indicated that 100-SW in this RHL population is regulated by *GmDt1*. This finding differs from most previous mapping studies on 100-SW, which generally reported regulation by multiple quantitative trait loci (QTLs) (Li Y. et al., 2024). This discrepancy may be due to the multiple generations of selfing in RHLs, which lead to homozygosity of non-target traits and minor-effect loci, while retaining only a few major-effect loci. Consequently, *GmDt1* acts as a major QTL in this specific genetic background. Such single-gene regulation is relatively common when mapping of 100-SW genes using soybean mutants. For example, progeny from the cross between the sss1 mutant and its wild-type parent ZP661 showed a single-gene regulatory pattern, and *GmSSS1* was identified as the candidate gene through fine mapping (Zhu et al., 2022). Therefore, the 100-SW regulatory gene identified in this study can be inferred as a major-effect candidate gene.

### 4.2 Analysis of candidate genes for soybean 100-SW

BSA-seq is a cost-effective strategy for primary QTL detection, but it usually results in relatively large genomic intervals. Further fine mapping or GWAS is typically required to narrow down the target region. In the present study, BSA-seq was employed to map the gene regulating soybean 100-SW, which was initially localized to a 5.46 Mb interval on chromosome 19. This result is supported by numerous previous studies focusing on genetic loci associated with the 100-SW trait. Multiple QTLs (seed weight 3-5; seed weight 7-7; seed weight 12-3; seed weight 13-9; seed weight 17-1; seed weight 42-4) on chromosome 19 have been consistently reported to be closely linked to soybean 100-SW (Mian et al., 1996; Orf et al., 1999; Csanádi et al., 2001; Hoeck et al., 2003; Stombaugh et al., 2004; Wang X. et al., 2015). Collectively, these pieces of evidence confirm that this region of chromosome 19 is a conserved key segment involved in regulating soybean 100-SW. This finding validates the reliability of our initial mapping result.

To identify the causal gene within this 5.46 Mb interval, further bioinformatic analysis and functional annotation suggested that *GmDt1* is a promising candidate gene for 100-SW regulation. As a well-characterized hub gene in soybean, *GmDt1* plays a crucial role in modulating podding habit and plant height (Liu et al., 2010; Tian et al., 2010). Notably, a recent study by Li Y. et al. (2024) demonstrated that *GmDt1* interacts with *GmSWEET10a* to coordinately modulate soybean seed size. This finding directly corroborates our results and expands our understanding of *GmDt1*’s functional versatility beyond plant architecture control.

To further decipher the molecular mechanism underlying *GmDt1*-mediated regulation of 100-SW, we analyzed its sequence variation and genetic pattern. Four non-synonymous mutations have been previously reported in *GmDt1* (Tian et al., 2010). Our study identified a novel natural variation located in the first exon of *GmDt1*. This variation is distinct from that reported by Li Y. et al. (2024), which was situated in the fourth exon, indicating a new functional locus contributing to soybean 100-SW regulation. In the present study, non-synonymous SNPs in coding regions were prioritized during candidate gene screening, as they are more likely to affect protein function. Variations in non-coding regions warrant further investigation in future studies. Additionally, genetic analysis revealed that *GmDt1* exerts differential effects on 100-SW in dominant homozygous and heterozygous genotypes, displaying an incomplete dominance inheritance pattern. This genetic characteristic is consistent with that of *GmSSS1*, another well-characterized 100-SW regulatory gene in soybean (Zhu et al., 2022). These findings suggest a conserved genetic regulatory mechanism for soybean 100-SW among these genes.

The RHL population used in this study is characterized by large seeds with determinate podding habit and small seeds with a semi-determinate podding habit. Investigation of agronomic traits in several lines from the ZP661 × ZH13 RIL population revealed that, although the population generally displayed the tendency of large seeds associated with dwarf plants and fewer nodes, and small seeds associated with tall plants and more nodes, some exceptional lines exhibited tall stature, more nodes, and large seeds simultaneously (**Supplementary Table 4**). Furthermore, although most cultivated soybean varieties carrying the *GmDt1-H2* haplotype show a determinate podding habit, those with a semi-erect stem growth habit still maintain relatively high 100-SW, such as ZH55 (26 g) and ZH74 (25.7 g) (**Supplementary Table 3**). Such incomplete linkage among 100-SW, podding habit, and plant height is highly valuable for breeding elite soybean varieties adapted to diverse ecological regions. Collectively, these results verify the key role of *GmDt1* in regulating soybean 100-SW and identify a novel functional variant. This finding lays a solid foundation for further functional characterization and molecular breeding applications.

### 4.3 The role of *GmDt1* gene in domestication and improvement of soybean 100-SW

Cultivated soybean (*Glycine max*) was domesticated from wild soybean (*Glycine soja*) (Asaf et al., 2017). During domestication, humans preferred larger seeds due to their higher yield potential (Zhu et al., 2022). Almost all wild soybeans harbor the small 100-SW haplotype *GmDt1-H1*. As humans selected varieties, other haplotypes emerged in landraces and improved cultivars. Notably, the high 100-SW haplotype *GmDt1-H2* identified in this study absent in wild soybeans was detected at a frequency of 9.07% in landraces and increased to 15.83% in improved cultivars. Although *GmDt1-H2* has not yet become the dominant haplotype, its gradual enrichment indicates that this elite haplotype will continue to be favored during crop improvement. Similar selection patterns have been observed for elite haplotypes of other seed-related genes, including *GmSW16.1*, *POWR1*, *GmST05*, and *GmCYP82C4* (Duan et al., 2022; Goettel et al., 2022; Chen et al., 2023; Li et al., 2024).

## 5 Conclusion

This study mapped a core candidate gene *GmDt* controlling 100-SW to chromosome 19 using BSA-seq technology. A G-to-T non-synonymous mutation in the first exon was confirmed as the functional locus driving 100-SW phenotypic variation. This represents a novel functional variant distinct from previously reported mutations in *GmDt1*. Whole-genome resequencing classified *GmDt1* into five distinct haplotypes. Notably, *GmDt1-H2* has undergone gradual artificial selection during the domestication transition from wild to cultivated soybean. Together, these results validate the critical role of *GmDt1* in 100-SW regulation and provide a valuable molecular target for improving soybean seed traits and enhancing yield potential in molecular breeding programs.

## Data Availability

The original contributions presented in the study are included in the article/**Supplementary Material**. Further inquiries can be directed to the corresponding author.

## Supplementary material

Supplementary Table 1 BLAST analysis of candidate gene protein sequences.

Supplementary Table 2 *GmDt1* gene variation loci and phenotypic analysis.
