# Supplementary material for: Natural variation of the GmDt1 gene affects the 100-seed weight of soybean
Source: Front Plant Sci. 2026 Apr 2;17:1801920. doi: 10.3389/fpls.2026.1801920 (PMC13083091; doi:10.3389/fpls.2026.1801920)
Supplement: Supplementary file 1 [file DataSheet1.docx]

Table S1 BLAST analysis of candidate gene protein sequences.

| Gene ID | Variation type | Gene function |
| --- | --- | --- |
| *Glyma.19G181900* | Non-synonymous mutation | Auxin response factors (ARFs) with a B3 DNA-binding domain (B3) |
| *Glyma.19G184850* | Loss of the start codon | No reports have been documented |
| *Glyma.19G190300* | Non-synonymous mutation | DUF668 domain-containing protein of unknown function |
| *Glyma.19G191700* | Non-synonymous mutation | Heat shock transcription factor (HSF) |
| *Glyma.19G191800* | Non-synonymous mutation | pre-mRNA cleavage complex |
| *Glyma.19G191900* | Non-synonymous mutation | pre-mRNA cleavage complex |
| *Glyma.19G192000* | Non-synonymous mutation | Related to glycoprotein synthesis |
| *Glyma.19G192100* | Non-synonymous mutation | No reports have been documented |
| *Glyma.19G192200* | Non-synonymous mutation | No reports have been documented |
| *Glyma.19G192500* | Non-synonymous mutation | Flower-tissue-specific expressed gene |
| *Glyma.19G192900* | Non-synonymous mutation | ATP-binding cassette (ABC) transporter, specifically expressed in roots, root tips, and nodules |
| *Glyma.19G193100* | Non-synonymous mutation | Protein kinase-related |
| *Glyma.19G193800* | Non-synonymous mutation | AN1-type zinc finger protein, root-tissue-specific expressed gene |
| *Glyma.19G193900* | Non-synonymous mutation | Purple acid phosphatase (PAP), specifically expressed in flowers |
| *Glyma.19G194300* | Non-synonymous mutation | Podding habit, *Dt1* |
| *Glyma.19G200000* | Non-synonymous mutation | Glycosyltransferase, root-tissue-specific expressed gene |
| *Glyma.19G200100* | Non-synonymous mutation | Related to protein chromosomal transmission, root-tissue-specific expressed gene |
| *Glyma.19G200300* | Non-synonymous mutation | DOF zinc finger frotein |
| *Glyma.19G200800* | Non-synonymous mutation | Nuclear transcription factor, root-tissue-specific expressed gene |
| *Glyma.19G200900* | Non-synonymous mutation | Pentanediol-related protein |
| *Glyma.19G201100* | Non-synonymous mutation | TRAF-LIKE family protein, root-tissue-specific expressed gene |
| *Glyma.19G201300* | Non-synonymous mutation | Polyketide cyclase/dehydratase and lipid transport superfamily protein |
| *Glyma.19G201400* | Non-synonymous mutation | Serine/threonine protein kinase (STK), root-tissue-specific expressed gene |
| *Glyma.19G201800* | Non-synonymous mutation | Zinc finger protein |
| *Glyma.19G202400* | Non-synonymous mutation | Leaf-tissue-specific expressed gene |
| *Glyma.19G203400* | Non-synonymous mutation | Glutamate carboxypeptidase |
| *Glyma.19G203500* | Non-synonymous mutation | Leaf-specific expressed gene |
| *Glyma.19G203800* | Non-synonymous mutation | Unknown function |
| *Glyma.19G203900* | Non-synonymous mutation | Protein containing TRAF, MATH, and BTB/POZ domains, leaf-tissue-specific expressed gene |
| *Glyma.19G204200* | Non-synonymous mutation | Cleavage and polyadenylation specificity factor (CPSF) |
| *Glyma.19G205200* | Non-synonymous mutation | Nuclear protein localization, root-tissue-specific expressed gene |
| *Glyma.19G205300* | Non-synonymous mutation | Catalase (CAT) |
| *Glyma.19G205400* | Non-synonymous mutation | Catalase (CAT) |
| *Glyma.19G205800* | Non-synonymous mutation | Catalase (CAT) |
| *Glyma.19G206000* | Non-synonymous mutation | Leaf-tissue-specific expressed gene |
| *Glyma.19G206100* | Non-synonymous mutation | Auxin response factor (ARF) |

Table S2 *GmDt1* gene variation loci and phenotypic analysis.

| Variety name | Variation type | 100-seed weight (g) |
| --- | --- | --- |
| ZH13 | T/T | 26.20 ± 0.96 |
| ZP661 | G/G | 19.75 ± 1.02 |
| ZH13-like-1 | T/T | 27.43 ± 1.33 |
| ZH13-like-2 | T/T | 26.32 ± 0.88 |
| ZH13-like-3 | T/T | 25.14 ± 1.67 |
| ZH13-like-4 | T/T | 25.97 ± 1.38 |
| ZH13-like-5 | T/T | 24.73 ± 0.97 |
| ZH13-like-6 | T/T | 24.68 ± 0.62 |
| ZH13-like-7 | T/T | 25.38 ± 2.01 |
| ZH13-like-8 | T/T | 28.20 ± 1.94 |
| ZH13-like-9 | T/T | 26.51 ± 1.67 |
| ZH13-like-10 | T/T | 26.30 ± 2.62 |
| ZH13-like-11 | T/T | 26.25 ± 1.36 |
| ZH13-like-12 | T/T | 27.57 ± 1.33 |
| ZP661-like-1 | G/G | 21.09 ± 2.42 |
| ZP661-like-2 | G/G | 19.35 ± 1.05 |
| ZP661-like-3 | G/G | 19.75 ± 1.01 |
| Intermediate-1 | G/T | 23.99 ± 0.67 |
| ZP661-like-4 | G/G | 18.55 ± 0.59 |
| ZP661-like-5 | G/G | 16.66 ± 1.25 |
| ZP661-like-6 | G/G | 21.54 ± 1.52 |
| Intermediate-2 | G/T | 24.85 ± 1.49 |
| Intermediate-3 | G/T | 23.83 ± 1.83 |
| ZP661-like-7 | G/G | 20.42 ± 0.78 |
| ZP661-like-8 | G/G | 21.20 ± 2.12 |
| Intermediate-4 | G/T | 22.83 ± 1.79 |
| ZP661-like-9 | G/G | 20.60 ± 0.98 |
| Intermediate-5 | G/T | 25.07 ± 1.11 |
| Intermediate-6 | G/T | 23.68 ± 1.26 |

**Fig. S1** Schematic diagram of the origin of the residual heterozygous line (RHL) with segregation for 100-seed weight


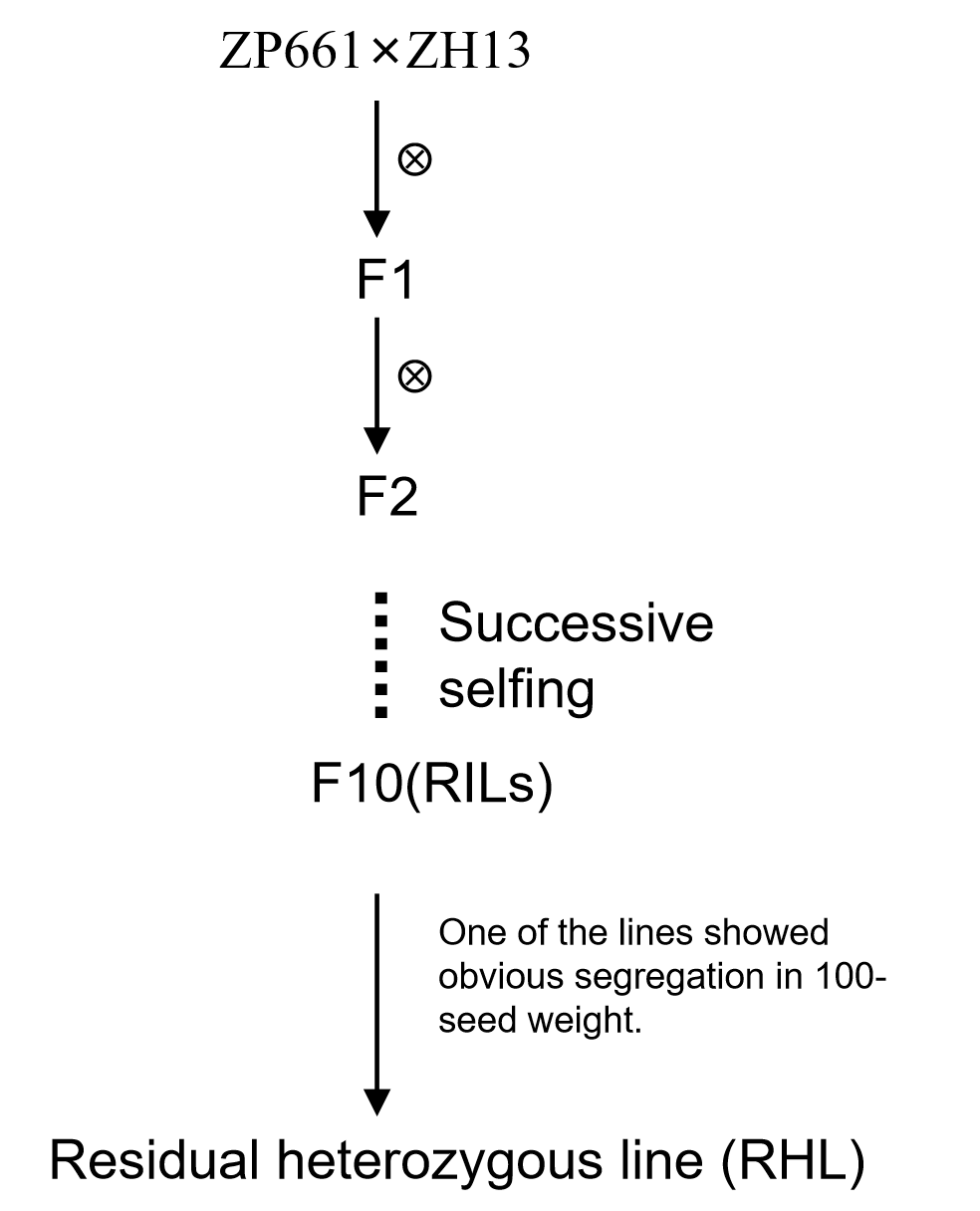


**Fig. S2** The marker validation of candidate gene *Glyma.19G181900*.


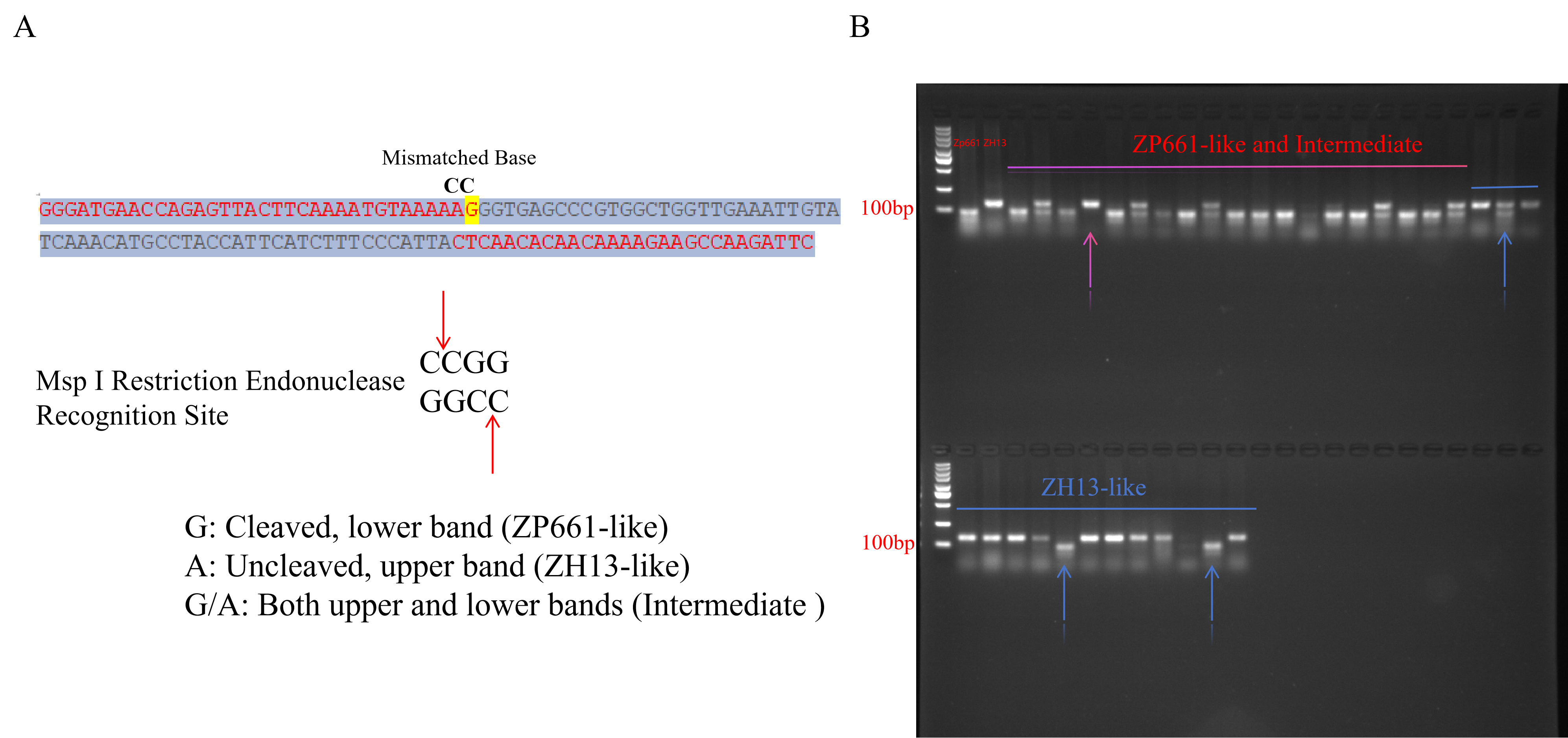


1. Schematic diagram of the restriction enzyme cutting site identified by dCAPS labeling; the red vertical line indicates the core restriction site. (B) dCAPS marker electrophoresis profile of the segregating population; arrows indicate individual plants with unlinked genotypic and phenotypic inheritance, and the marker is 5000 bp.
